# Characteristics of a Three-Generation Family with Stickler Syndrome Type I Carrying Two Different COL2A1 Mutations

**DOI:** 10.3390/genes14040847

**Published:** 2023-03-31

**Authors:** Adam Jacobson, Cagri G. Besirli, Brenda L. Bohnsack

**Affiliations:** 1 Kellogg Eye Center, Department of Ophthalmology and Visual Sciences, University of Michigan, Ann Arbor, MI 48105, USA; 2 Division of Ophthalmology, Ann & Robert H. Lurie Children’s Hospital of Chicago, Chicago, IL 60611, USA; 3 Department of Ophthalmology, Feinberg School of Medicine, Northwestern University, Chicago, IL 60208, USA

**Keywords:** COL2A1, Stickler syndrome, vitreoretinal degeneration

## Abstract

Stickler Syndrome is typically characterized by ophthalmic manifestations including vitreous degeneration and axial lengthening that predispose to retinal detachment. Systemic findings consist of micrognathia, cleft palate, sensorineural hearing loss, and joint abnormalities. COL2A1 mutations are the most common, however, there is a lack of genotype-phenotype correlations. Retrospective, single-center case series of a three-generation family. Clinical features, surgical requirements, systemic manifestations, and genetic evaluations were collected. Eight individuals clinically displayed Stickler Syndrome, seven of whom had genetic confirmation, and two different COL2A1 mutations (c.3641delC and c.3853G>T) were identified. Both mutations affect exon 51, but display distinct phenotypes. The c.3641delC frameshift mutation resulted in high myopia and associated vitreous and retinal findings. Individuals with the c.3853G>T missense mutation exhibited joint abnormalities, but mild ocular manifestations. One individual in the third generation was biallelic heterozygous for both COL2A1 mutations and showed ocular and joint findings in addition to autism and severe developmental delay. These COL2A1 mutations exhibited distinct eye vs. joint manifestations. The molecular basis for these phenotypic differences remains unknown and demonstrates the need for deep phenotyping in patients with Stickler syndrome to correlate COL2A1 gene function and expression with ocular and systemic findings.

## 1. Introduction

Stickler Syndrome is a collagenopathy affecting numerous organ systems, most commonly ocular, auditory, and musculoskeletal [1,2,3]. It is categorized into types I-V, based on the collagen gene that is mutated. Stickler Syndrome type I (STL1, OMIM 108300) is most common, responsible for 70–90% of cases, and is due to autosomal dominant mutations in the COL2A1 gene located on chromosome 12 [1,3]. Patients with Stickler Syndrome type I typically present with high myopia with vitreoretinal degeneration, hypermobility with osteoarthritis, and hearing impairment. Furthermore, patients often present with the Pierre Robin sequence, which is characterized by micrognathia, glossoptosis, and cleft palate [1,2,3].

Stickler Syndrome as one of many syndromes associated with COL2A1 mutations, presents with a broad range of anatomical findings and ophthalmological complications, often leading to difficulty with diagnosis, treatment, and genetic counseling [4]. Recent reviews have begun to detail these varied presentations with genotype-phenotype correlations as technology continues to progress [5].

Autosomal dominant and recessive inheritance patterns associated with different target genes (COL2A1, COL11A1, COL11A2, COL9A1, and COL9A3) have been identified [1,3,5,6,7]. Most patients with autosomal dominant Stickler Syndrome carry one mutated collagen allele, however, there has been one reported case of biallelic heterozygosity of COL11A1 mutations which resulted in retinal atrophy and severe hearing loss [8]. 

The COL2A1 gene encodes for α-1 procollagen type II, which is the integral protein for type II collagen. Following trimerization and cleavage of the pro-peptides, mature type II collagen forms a necessary fibrillar network within the extracellular matrix of cartilage, the vitreous, and intervertebral discs [9,10,11,12,13]. While there are many diseases that are associated with mutations in COL2A1, Stickler syndrome, an arthro-ophthalmopathy, is most common [3,4,5,12,13]. Predominantly characterized by the ocular findings, namely vitreoretinal degeneration and pathologic myopia often complicated by retinal tears and detachments, systemic findings vary but include micrognathia, cleft palate, early osteoarthritis and joint problems, and deafness [2,3,4,5,12,13]. 

The pathogenesis of eye findings in Stickler syndrome is not well defined. In adults, COL2A1 is expressed in the vitreous [14]. This likely accounts for the characteristic vitreous degeneration seen in Stickler syndrome, which can cause traction leading to retinal tears and detachments. However, the basis for axial lengthening and high myopia, which predisposes to retinal pathologies, remains unknown. Unlike the majority of newborns who are hyperopic, individuals with Stickler syndrome typically show moderate to high myopia at birth [15]. Furthermore, the association between Stickler syndrome and infantile-onset glaucoma suggests that type II collagen is important in the development of the sclera and aqueous humor outflow system [14,15,16]. Infantile onset glaucoma associated with Stickler syndrome is a likely underreported disease process with few case reports [14]. Like the complex retinal detachments often seen in association with Stickler syndrome, the associated secondary glaucoma is often difficult to treat. In the largest case report of pediatric patients with STL1 and secondary infantile-onset glaucoma, all patients required multiple intraocular pressure lowering surgeries and three eyes became phthisical in the presence of a retinal detachment [14]. 

A mouse model confirmed Col2a1 expression in the vitreous, but also showed weaker expression in the post-natal cornea, ciliary body, iris, lens, retina, choroid, and sclera. [17] Col2a1 was down-regulated in Col2a1 +/− adult mice in all of these tissues, although the function of type II collagen especially within the developing sclera and anterior segment has yet to be defined. Additional studies are required to provide insight into the pathogenesis of the ocular findings associated with COL2A1 mutations. 

**Figure 1:**
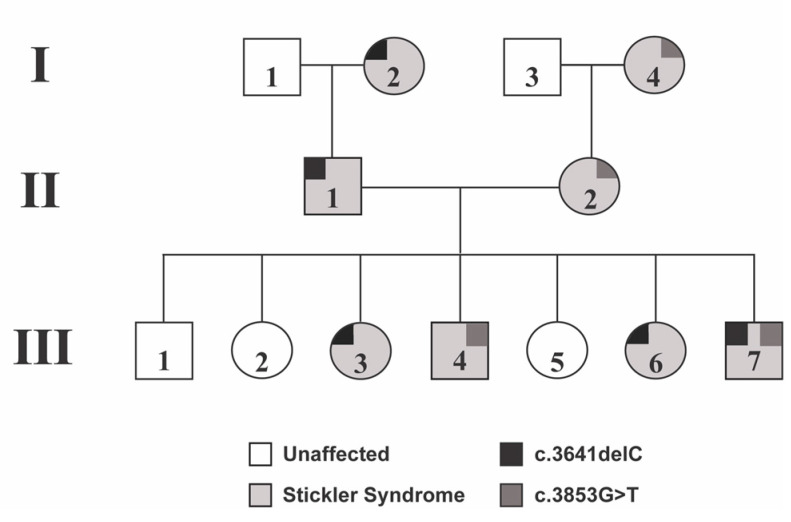
Three-generation pedigree. A pedigree showing affected and non-affected family members, along with specific mutations within COL2A1. Of note, Patient III-7 carries both maternally and paternally inherited mutation.

We present a three-generation pedigree of patients with Stickler Syndrome type I with two different COL2A1 mutations (Figure 1). The third generation includes a sibling group of seven, four of whom have Stickler Syndrome type I and display a combination of maternally inherited and paternally inherited COL2A1 mutations. This pedigree allows us to better analyze phenotypes associated with these COL2A1 mutations (Table 1), and to the best of our knowledge, we report the first patient with biallelic COL2A1 heterozygous mutations. 

## 2. Case Description

This study was approved by the University of Michigan Institutional Review Board and the research was performed in accordance with the Declaration of Helsinki. A retrospective chart review was performed on members of the family of interest. Date of birth, ophthalmic and systemic diagnoses, ophthalmic history, medical history, and surgical history were recorded. Clinical findings were collected, including best corrected visual acuity (BCVA), intraocular pressure (IOP), manifest and/or cycloplegic refraction, axial length, slit lamp exam, dilated fundus exam, and surgical requirements and outcomes. 

Visual acuity was determined by Snellen optotype acuity when possible. In one non-verbal patient, non-optotype acuity was documented as “fix and follow” and the presence of a fixation preference was determined by an induced tropia test. Manifest refraction at a phoropter was performed on all adult patients, and cycloplegic retinoscopy was performed on all pediatric patients. Axial length was determined by immersion A-scan on all patients, under general anesthesia if necessary. Intraocular pressure was measured by Icare (Revenio, Vantaa, Finland), Tono-pen (Reichert, Depew, NY, USA), or Goldmann applanation. All patients underwent genetic testing with targeted COL2A1 sequencing by CLIA-certified Connective Tissue Gene Tests (Allentown, PA, USA) protocol via Sanger sequencing on ABI3730 sequencers (Thermofisher, Waltham, MA, USA).

### 2.1. Patient II-1

Patient II-1 was a 48-year-old man who had previously been diagnosed with Stickler Syndrome type I both clinically and genetically. He had a history of retinal detachment of the right eye, which required surgical repair at an outside institution. Genetic testing revealed a frameshift mutation in COL2A1 (NM_001844.5, c.3641delC) that was also carried by his mother (Patient I-2). By report, Patient II-1’s mother had a history of multiple retinal detachments, but additional information was unavailable. Patient II-1 had a history of essential hypertension and acute cholecystitis, which required cholecystectomy. On initial examination, Patient II-1 had hand motion vision in the right eye and best corrected visual acuity of 20/40 in the left. He was moderately myopic in both eyes and axial lengths were 25.38 mm and 25.45 mm in the right and left eyes, respectively. Intraocular pressures and anterior segment slit lamp exam were unremarkable other than nuclear sclerotic cataracts. Fundus examination of the right eye showed chorioretinal scarring superiorly, numerous foci of radially oriented lattice degeneration, five peripheral horseshoe tears, and old vitreous hemorrhage inferiorly. The left eye showed areas of chorioretinal scarring superiorly and inferiorly without breaks or tears. Patient II-1 underwent combined cataract extraction with an intraocular lens implant (CEIOL) and laser retinopexy in his right eye. His best corrected visual acuity immediately improved to 20/100 until five weeks post-operatively when he presented with a macula-off rhegmatogenous retinal detachment. He underwent a pars plana vitrectomy with endolaser, and gas tamponade with perfluoropropane. His right eye eventually improved to a best-corrected visual acuity of 20/60 and he has not required surgery in his left eye.

### 2.2. Patient II-2

Patient II-2 was a 49-year-old woman with a known diagnosis of Stickler Syndrome type I who had not required previous ocular surgery. Genetic testing showed a missense mutation in COL2A1 (NM_001844.5, c.3853G>T) that was notably different from her husband’s (Patient II-1). By history, her mother (Patient I-4) had Stickler Syndrome with a history of retinal detachment, but no genetic information or additional clinical details were available. Patient II-2 had a history of multiple tooth abscesses and polyarticular early-onset osteoarthritis requiring bilateral total hip arthroplasties. Initial examination showed 20/25 best corrected visual acuity in the right eye and count fingers acuity in the left eye. Her manifest refraction was −6.00 + 2.50 × 078 in the right eye and −5.50 + 1.25 × 073 in the left eye. Axial lengths were 24.82 mm and 25.39 mm in the right and left eyes, respectively. Intraocular pressure and pupil exam were normal. Anterior and posterior exam in the right eye was unremarkable. The left eye showed a mature cataract with no view to the posterior segment. B scan ultrasonography in the left eye showed vitreous opacities with a posterior vitreous detachment without signs of a retinal detachment. Following cataract extraction with an intraocular lens implant, the patient’s best corrected visual acuity in the left eye was 20/50 and to date, neither eye has required retinal interventions. 

### 2.3. Patient III-3

Patient III-3 was a 18-year-old girl with high myopia and bilateral patellar instability. Genetic evaluation showed a heterozygous frameshift mutation in COL2A1 (c.3641delC) inherited from her father (Patient II-1). The patient also had generalized joint pain and tachycardia. At her initial examination, her best corrected visual acuity was 20/30 in the right eye and 20/40 in the left eye. Her cycloplegic refraction at that time was −14.00 + 2.00 × 105 in the right eye and −11.25 + 1.00 × 135 in the left eye. The slit lamp exam was unremarkable and her intraocular pressure was normal. Her fundus exam showed myopic appearing optic nerves with two isolated congenital hypertrophy of the retinal pigment epithelium (CHRPE) lesions in the right eye and scattered lattice degeneration in both eyes. Furthermore, she had a longstanding 30-prism diopter esotropia without diplopia.

After the initial visit, Patient III-1 underwent prophylactic indirect laser photocoagulation in both eyes in addition to bilateral medial rectus recessions for the esotropia. Four months following the strabismus surgery, her acuity decreased in the left eye to 20/125 along with a reported increase in floaters. Her examination was normal, and these changes were thought to be due to an impending posterior vitreous detachment. She was seen two months later and was noted to have a complete return of vision in the affected eye.

### 2.4. Patient III-4

**Figure 2:**
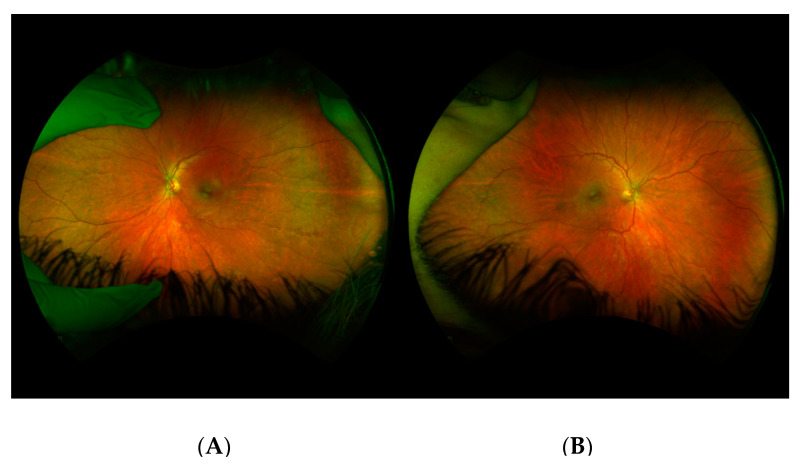
Fundus photos of Patient III-4. Fundus photos of Patient III-4 showing a normal posterior pole in the right eye (**A**) with a small CHRPE at 3:00 in the left eye (**B**).

Patient III-4 was a 17-year-old boy who presented due to his family history of Stickler Syndrome and a history of stiffness of both hip joints and multiple epiphyseal dysplasia. A genetic evaluation showed a frameshift mutation in COL2A1 (c.3853G>T), that was inherited from his mother (Patient II-2). At his initial examination, the best corrected visual acuity was 20/20 in both eyes. Cycloplegic refraction was −0.25 + 0.50 × 180 in the right eye and −0.75 + 1.25 × 180 in the left eye. The anterior segment exam and intraocular pressures were normal. Dilated fundoscopic examination showed mild vitreous syneresis and a small CHRPE in the left eye (Figure 2). He had not undergone any eye surgeries at the time of data collection.

### 2.5. Patient III-6

**Figure 3:**
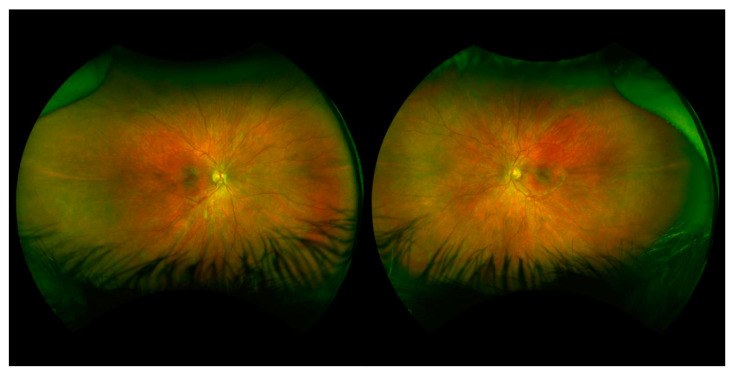
Fundus photos of Patient III-6. Fundus photos showing a tilted, myopic-appearing optic nerves and a myopic-appearing fundus.

Patient III-6 was a 9–year-old girl who presented due to her family history of Stickler Syndrome and high myopia. A genetic evaluation showed a frameshift mutation in COL2A1 (c.3641delC), which had been inherited from her father. At her initial examination, her best corrected visual acuity was 20/30 in the right eye and 20/40 in the left eye. Cycloplegic refraction was −11.50 + 2.00 × 85 in the right eye and −13.75 + 2.50 × 95 in the left eye. Her anterior segment and intraocular pressure were normal. Dilated fundoscopic examination showed vitreous syneresis and myopic and tilted optic nerves, but no evidence of retinal holes or detachments. Anterior and posterior exams were normal, other than vitreous syneresis and myopic appearing optic nerves (Figure 3). She had not undergone any eye surgeries at the time of data collection. 

### 2.6. Patient III-7

Patient III-7 was a 4-month-old boy who presented for an eye examination due to facial characteristics consistent with the Pierre Robin sequence and the family history of Stickler Syndrome. His genetic testing showed biallelic, heterozygous COL2A1 mutations, inherited from his mother (c.3853G>T) and father (c.3641delC). For his micrognathia and cleft palate, he underwent several rounds of mandibular and palate repair. He also had sensorineural hearing loss and Eustachian tube dysfunction and was status post bilateral cochlear implants and multiple myringotomies with tubes. The patient also had bilateral hip and femoral dysplasia, severe non-verbal autism, and global developmental delay. 

**Figure 4:**
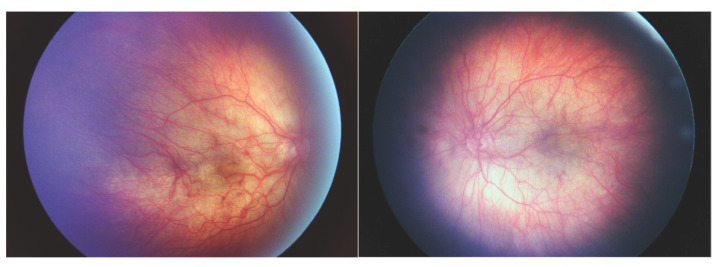
Fundus photos of Patient III-7. Fundus photos of Patient III-7 showing poor foveal reflex, mild vessel tortuosity, and peripheral pigmentation in the **right** (Figure 2A) and **left** (Figure 2B).

At his initial eye examination, the patient was able to fix and follow with each eye and showed no preference for induced tropia test. Cycloplegic refraction was –9.50 in the right eye and –8.50 in the left eye. Anterior segment examination and intraocular pressures were normal. Dilated fundus examination showed poor foveal reflex, mild vessel tortuosity, and peripheral pigmentation bilaterally (Figure 4). At 3 years of age, his myopia had progressed to −13.50 + 2.00 × 090 in the right eye and −15.50 + 3.00 × 090 in the left eye, and he underwent prophylactic peripheral laser treatment in both eyes. Axial lengths at that time were 28.69 mm and 29.13 mm in the right and left eyes, respectively. At the time of data collection, he has not developed retinal complications and has not required other eye surgeries.

## 3. Discussion

We present a three generation family with genotypic and phenotypic descriptions of two different COL2A1 mutations. Furthermore, we present an individual with biallelic, autosomal dominant, heterozygous Stickler Syndrome type I, resulting in severe ocular and systemic findings (Patient III-7).

The COL2A1 gene has 54 exons and the majority of the coding region (4464 base pairs) comprises the triple-helical domain that consists of approximately 300 Gly-X-Y repeats [4,11]. Mutations associated with Stickler Syndrome type I have been documented throughout the gene and vary in severity and phenotype [3,4,5,12]. Both mutations found in this family affected exon 51 with a single nucleotide deletion at 3641 and a missense mutation at 3853. Exon 51 is at the end of the triple helical domain and has commonly been associated with mild Stickler Syndrome type I. These mutations being close to the C terminus are likely less severe compared to mutations near the N terminus or at the beginning of the triple helix as the majority of the protein is intact [4,5]. 

**Figure 5:**
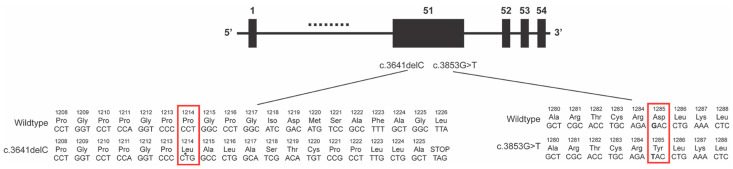
Effects of COL2A1 mutations in Exon 51 on protein sequence. Both the c3641delC and c.3853G>T mutations affect exon 51 in the COL2A1 gene. The c.3641 deletion from the wildtype sequence leads to a frameshift resulting in the change in sequence after Proline 1213 and a premature stop codon after Alanine 1225. The c.3853G>T mutation changes Aspartic Acid 1285 to a Tyrosine.

The c.3641delC mutation causes a frameshift that affects the last portion of the triple helix domain and the C terminus (Figure 5) [4]. The c.3641 locus had previously been reported in a disease-causing frameshift duplication, however, little phenotype information is included except for a binary logistic regression analysis score which is consistent with a moderate phenotype. [13] Of the two mutations in this report, the c.3641delC had more severe ocular phenotypes with the three affected monoallelic individuals (patients II-1, III-3, and III-6) having high myopia (higher than −11.00) and associated retinal findings such as retinal tears and lattice degeneration. In addition, patient II-1 was the only member in the second or third generations to develop a retinal detachment.

The 3853G>T missense mutation resulted in a p.Asp1285Tyr amino acid change within the triple helix domain (Figure 5). We had included this mutation in a prior report that included only patient III-7 as the rest of the family had not been examined at that time [15]. Otherwise, there have been no other reports of this locus, and our previous report included little specific phenotype information on this mutation. In contrast to the c.3641delC mutation, the 2 affected monoallelic individuals (patients II-2 and III-4) showed mild ocular phenotypes. On the other hand, both of these patients had joint abnormalities such that patient II-2 required bilateral hip arthroplasty for early onset osteoarthritis in her 40s and patient III-4 had multiple epiphyseal dysplasia. Isolated early-onset osteoarthritis has been considered its own COL2A1 entity and in the few reported cases is associated with a specific missense mutation much earlier in the coding region (c.823C>T) [18,19,20]. Additional COL2A1 mutations (c.2503C>T and c.2032G>A) have been identified in multiple epiphyseal dysplasia, with one family also having myopia and deafness [21,22]. Since multiple epiphyseal dysplasia is typically diagnosed radiologically and is clinically characterized by joint pain, stiffness, and early onset osteoarthritis, this may be under-diagnosed in Stickler Syndrome type I.

The joint and bone abnormalities associated with COL2A1 mutations vary from mild to severe. In the milder forms, as typically seen in Stickler Syndrome type I, joints are more affected than bones [4,5]. In these cases, the mutant type II collagen seems to be able to form fibrils, albeit abnormal, which leads to progressive degeneration [23]. In more severe forms, leading to skeletal dysplasia, the mutant type II collagen is unable to assemble into fibrils, resulting in endoplasmic reticulum-mediated chondrocyte apoptosis and extracellular matrix loss [24,25]. Furthermore, the paucity of type II collagen fibrils within the extracellular matrix inhibits chondrocyte differentiation which is necessary for bone formation [26]. 

Although both of these mutations affect exon 51, they display different Stickler Syndrome type I phenotypes, which is highlighted by their juxtaposition within this family. With the identification of disease-causing loci, Stickler Syndrome type I has been associated with missense and nonsense mutations as well as nucleotide deletions and duplications [4,12]. Furthermore, mutations affecting the N terminus, triple helix domain, and C terminus have all been reported [4,12]. Traditionally nonsense mutations and frameshift deletions and duplications have more deleterious effects than single nucleotide missense mutations due to the activation of nonsense-mediated mRNA decay and greater capacity for altering the protein sequence. However, a prior study by Zhang et al. found that phenotype severity is similar between nonsense, frameshift, and missense mutations. The phenotypes were graded as mild, severe, and lethal, but did not include specific information regarding ocular vs. joint manifestations [4]. Some phenotypic differences may also be due to differential expression of the two COL2A1 isoforms that result from alternative splicing near exon 2, which is in the N terminus [27]. The longer form (type IIA) is expressed during embryogenesis and in the adult vitreous. In contrast, the shorter form (type IIB) is found in adult chondrocytes [28]. However, as this family’s mutations are much further downstream in the COL2A1 gene, this is unlikely the reason for their phenotypic differences. Overall, there is a need for deep phenotyping in Stickler Syndrome type I to specifically correlate mutation types and loci to specific ocular and systemic findings.

As a result of distinct paternal and maternal mutations, one individual in the third generation had biallelic heterozygous COL2A1 mutations. One individual with two COL11A1 mutations in trans has been reported and exhibited retinal atrophy and severe hearing loss [8]. Furthermore, autosomal recessive COL9A3 mutations are also associated with Stickler syndrome [3,7]. However, to the best of our knowledge, we present the first documented case involving COL2A1. 

This patient displayed pathologic myopia and vitreoretinal degeneration like his father and two sisters who carry the c.3641delC mutation, previously described as a mutation resulting in such ophthalmic abnormalities [13,15]. Furthermore, he had dysplastic joints similar to his mother and brother who had the c.3853G>T mutation. He also had other systemic findings that are commonly associated with Stickler syndrome, but were not found in his other family members including micrognathia, cleft palate, and hearing loss. Additionally, this patient had neurological diagnoses (non-verbal autism and severe developmental delay) that are not typically seen in Stickler syndrome. While children with Stickler syndrome can have learning disabilities and behavior issues, these are typically attributed to their vision and hearing impairments. There is an unclear role for COL2A1 in brain development, thus it is unknown whether these atypical findings are due to his biallelic heterozygous COL2A1 mutations or other etiologies. 

## 4. Conclusions

In summary, we present a three-generation pedigree with Stickler Syndrome type I that highlights ocular vs. joint phenotypes associated with COL2A1 mutations. While both of the COL2A1 mutations that affect family members are located in the same exon, one is a frameshift nucleotide deletion and one is a missense mutation. The molecular basis for these phenotypic differences is unknown. More studies with deep phenotyping are needed to fully understand the phenotype-genotype correlations in Stickler syndrome.

## Figures and Tables

**Table 1:** Ocular and systemic characteristics in family members with Stickler syndrome type I Clinical findings of the eight patients with Stickler syndrome show the phenotypic variability with the family of interest, including ocular, systemic, and genetic findings.

Patient	Mutation	Myopia	Retina	Ocular Surgeries	Systemic Problems	Systemic Surgeries
I-2	c.3641delC (presumed)	unknown	RD × 2	unknown	unknown	unknown
I-4	c.3853G>T (presumed)	unknown	RD × 1	unknown	unknown	unknown
II-1	c.3641delC	unknown	OD: chorioretinal scarring, lattice, HRT × 5, VH, RD OS: chorioretinal scarring	CEIOL OD Laser OD PPV/EL OD	Hypertension Cholecystitis	Cholecystectomy
II-2	c.3853G>T	OD: −6.00 +2.75 OS: −3.00 + 0.75	OU: normal	CEIOL OS	Joint pain Osteoarthritis Tooth abscess	Right closed hip reduction Total right hip arthroplasty × 2 Total left hip arthroplasty
III-1	None	-	-	-	-	-
III-2	None	-	-	-	-	-
III-3	c.3641delC	OD: −14.00 +2.00 OS: −11.25 +1.00	OD: myopic nerve, CHRPE, lattice OS: lattice	Laser OU Strabismus	Joint pain Patellar instability Tachycardia	None
III-4	None	-	-	-	-	-
III-5	c.3853G>T	OD: −0.25 +0.50 OS: −0.75 +1.25	OD: normal OS: CHRPE	None	Hip joint stiffness Multiple epiphyseal dysplasia	None
III-6	c.3641delC	OD: −11.50 +2.00 OS: −13.75 +2.50	OD: myopic nerve OS: myopic nerve	None	None	None
III-7	c.3641delC c.3853G>T	OD: −13.50 +2.00 OS: −15.50 +3.00	Poor foveal reflex Mild vessel tortuosity Peripheral pigmentation	Laser OU	Pierre Robin sequence Cleft palate Micrognathia Femoral head dysplasia Bilateral hip dysplasia Sensorineural hearing loss Eustachian tube dysfunction Autism Non-verbal Developmental delay	Cholecystectomy

RD: retinal detachment; Laser: prophylactic indirect laser photocoagulation; CHRPE: congenital hypertrophy of the retinal pigment epithelium; Lattice: lattice degeneration; OD: right eye; OS: left eye; OU: both eyes; CEIOL: cataract extraction with intraocular lens implant; HRT: horseshoe retinal tear; VH: vitreous hemorrhage; PPV: pars plana vitrectomy; EL: endolaser.

## Data Availability

Data can be requested by contacting the corresponding author.

## References

[1] Snead M.P., Yates J.R. (1999). Clinical and molecular genetics of Stickler syndrome. J. Med. Genet..

[2] Stickler G.B., Hughes W., Houchin P. (2001). Clinical features of hereditary progressive arthro-ophthalmopathy (*Stickler syndrome*): A survey. Genet. Med..

[3] Boothe M., Morris R., Robin N. (2020). Stickler syndrome: A review of clinical manifestations and the genetics evaluation. J. Pers. Med..

[4] Zhang B., Zhang Y., Wu N., Li J., Liu H., Wang J. (2019). Integrated analysis of *COL2A1* variant data and classification of type II collagenopathies. Clin. Genet..

[5] Soh Z., Richards A.J., McNinch A., Alexander P., Martin H., Snead M.P. (2022). Dominant Stickler Syndrome. Genes.

[6] Majava M., Hoornaert K.P., Bartholdi D., Bouma M.C., Bouman K., Carrera M., Devriendt K., Hurst J., Kitsos G., Niedrist D. (2007). A report on 10 new patients with heterozygous mutations in the *COL11A1* gene and a review of genotype-phenotype correlations in type XI collagenopathies. Am. J. Med. Genet. A.

[7] Markova T., Sparber P., Borovikov A., Nagornova T., Dadali E. (2021). Clinical and genetic characterization of autosomal recessive Stickler syndrome caused by novel compound mutations in the COL9A3 gene. Mol. Genet. Genomic Med..

[8] Nixon T., Richards A.J., Lomas A., Abbs S., Vasudevan P., McNinch A., Alexander P., Snead M.P. (2020). Inherited and de novo biallelic pathogenic variants in COL11A1 result in type 2 Stickler syndrome with severe hearing loss. Mol. Genet. Genomic Med..

[9] Strom C.M., Upholt W.B. (1984). Isolation and characterization of genomic clones corresponding to the hujman type II procollagen gene. Nucleic Acids Res..

[10] Bella J., Hulmes D.J. (2017). Fibrillar collagens. Subcell Biochem..

[11] Brodsky B., Persikov A.V. (2005). Molecular structure of the collagen triple helix. Adv. Protein Chem..

[12] Wang D.-D., Gao F.-J., Hu F.-Y., Zhang S.-H., Xu P., Xu J.-H. (2020). Mutation spectrum of Stickler syndrome type I and genotype-phenotype analysis in East Asian population: A systematic review. BMC Med. Genet..

[13] Hoornaert K.P., Vereecke I., Dewinter C., Rosenberg T., Beemer F.A., Leroy J.G., Bendix L., Björck E., Bonduelle M., Boute O. (2010). Stickler syndrome caused by COL2A1 mutations: Genotype-phenotype correlation in a series of 100 patients. Eur. J. Hum. Genet..

[14] Kashiwagi Y., Nishitsuka K., Takamura H., Yamamoto T., Yamashita H. (2011). Cloning and characterization of human vitreous-tissue derived cells. Acta Ophthalmol..

[15] Wubben T.J., Branham K.H., Besirli C.G., Bohnsack B.L. (2018). Retinal detachment and infantile-onset glaucoma in Stickler syndrome associated with known and novel COL2A1 mutations. Ophthalmic Genet..

[16] Ziakas N.G., Ramsay A.S., Lynch S.A., Clarke M.P. (1998). Sticklers syndrome associated with congential glaucoma. Ophthalmic Genet..

[17] Kaarniranta K., Ihanamäki T., Sahlman J., Pulkkinen H., Uusitalo H., Arita M., Tammi R., Lammi M.J., Helminen H.J. (2006). A mouse model for Stickler’s syndrome: Ocular phenotype of mice carrying a targeted heterozygous inactivation of type II (pro) collagen gene (Col2a1). Exp. Eye Res..

[18] Williams C.J., Considine E.L., Knowlton R.G., Reginato A., Neumann G., Harrison D., Buxton P., Jimenez S., Prockop D.J. (1993). Spondyloepiphyseal dysplasia and precocious osteoarthritis in a family with an Arg_75_→Cys mutation in the procollagen type II gene (COL_2_A_1_). Hum. Genet..

[19] Löppönen T., Körkkö J., Lundan T., Seppänen U., Ignatius J., Kääriäinen H. (2004). Childhood-onset osteoarthritis, tall stature, and sensorineural hearing loss associated with Arg_75_-Cys mutation in procollagen type II gene (COL_2_A_1_). Arthritis Rheum..

[20] Hoornaert K.P., Dewinter C., Vereecke I., Beemer F.A., Courtens W., A Fryer A., Fryssira H., Lees M., Müllner-Eidenböck A., Rimoin D.L. (2006). The phenotypic spectrum in patients with arginine to cysteine mutations in the COL2A1 gene. J. Med. Genet..

[21] Ballo R., Beighton P.H., Ramesar R.S. (1998). Stickler-like syndrome due to a dominant negative mutation in the COL2A1 gene. Am. J. Med. Genet..

[22] Dasa V., Eastwood J.R.B., Podgorski M., Park H., Blackstock C., Antoshchenko T., Rogala P., Bieganski T., Jazwinski M.S., Czarny-Ratajczak M. (2019). Exome sequencing reveals a novel COL2A1 mutation implicated in multiple epiphysela dysplasia. Am. J. Med. Genet. A.

[23] MacRae M.E., Patel D.V., Richards A.J., Snead M.P., Tolmie J., Lee W.R. (2005). Type 1 Stickler syndrome: A histological and ultrastructural study of an untreated globe. Eye.

[24] Chung H.J., Jensen D.A., Gawron K., Steplewski A., Fertala A. (2009). R992C (p.R1192C) Substitution in collagen II alters the structure of mutant molecules and induces the unfolded protein response. J. Mol. Biol..

[25] Liang G., Lian C., Huang D., Gao W., Liang A., Peng Y., Ye W., Wu Z., Su P., Huang N. (2014). Endoplasmic reticulum stress-unfolding protein response-apoptosis cascade causes chondrodysplasia in a col2a1 p.Gly1170Ser mutated mouse model. PLoS ONE.

[26] Barbieri O., Astigiano S., Morini M., Tavella S., Schito A., Corsi A., Di Martino D., Bianco P., Cancedda R., Garofalo S. (2003). Depletion of cartilage collagen fibrils in mice carrying a dominant negative Col2a1 transgene affects chondrocyte differentiation. Am. J. Physiol. Cell Physiol..

[27] McAlinden A., Majava M., Bishop P.N., Perveen R., Black G.C., Pierpont M.E., Ala-Kokko L., Männikkö M. (2008). Missense and nonsense mutations in the alternatively-spliced exon 2 of COL2A1 cause the ocular variant of Stickler syndrome. Hum. Mutat..

[28] McAlinden A. (2014). Alternative splicing of type II procollagen: IIB or not IIB?. Connect. Tissue Res..

